# Neural Correlates of Math Gains Vary Depending on Parental Socioeconomic Status (SES)

**DOI:** 10.3389/fpsyg.2016.00892

**Published:** 2016-06-17

**Authors:** Özlem Ece Demir-Lira, Jérôme Prado, James R. Booth

**Affiliations:** ^1^Department of Communication Sciences and Disorders, Northwestern University, EvanstonIL, USA; ^2^Department of Psychology, University of Chicago, ChicagoIL, USA; ^3^Institut des Sciences Cognitives Marc Jeannerod, UMR 5304, Centre National de la Recherche Scientifique – Université de LyonBron, France; ^4^Department of Communication Sciences and Disorders, The University of Texas at Austin, AustinTX, USA

**Keywords:** socioeconomic status, arithmetic, subtraction, fMRI, longitudinal, children

## Abstract

We used functional magnetic resonance imaging (fMRI) to examine the neural predictors of math development, and asked whether these predictors vary as a function of parental socioeconomic status (SES) in children ranging in age from 8 to 13 years. We independently localized brain regions subserving verbal versus spatial processing in order to characterize relations between activation in these regions during an arithmetic task and long-term change in math skill (up to 3 years). Neural predictors of math gains encompassed brain regions subserving both verbal and spatial processing, but the relation between relative reliance on these regions and math skill growth varied depending on parental SES. Activity in an area of the left inferior frontal gyrus (IFG) identified by the verbal localizer was related to greater growth in math skill at the higher end of the SES continuum, but lesser improvements at the lower end. Activity in an area of the right superior parietal cortex identified by the spatial localizer was related to greater growth in math skill at the lower end of the SES continuum, but lesser improvements at the higher end. Results highlight early neural mechanisms as possible neuromarkers of long-term arithmetic learning and suggest that neural predictors of math gains vary with parental SES.

## Introduction

Children from disadvantaged backgrounds as a group fall behind their peers in math achievement and math skill growth starting from the early grades (Pungello et al., 1996; Cheadle, 2008; National Center for Education Statistics, 2011). However, some of the children from disadvantaged backgrounds exhibit developmental trajectories that are similar to their peers from more advantaged backgrounds. Whether these children recruit the same neural systems as their peers or recruit alternative systems is not known. In the present study, we used functional magnetic resonance imaging (fMRI) to examine the neural predictors of long-term change in children’s math skill, and asked whether these predictors vary as a function of parental socioeconomic status (SES). Identifying early predictors of math skill growth in children from varying backgrounds might aid our understanding of the reasons behind individual differences in math skill growth. Increased understanding of the mechanisms behind these individual differences in turn might have implications for decreasing the achievement gap.

Mathematics is built upon earlier developing, existing verbal and spatial skills (Dehaene et al., 1999). In solving arithmetical problems, adults and children rely upon a wide network of brain regions, including regions that underlie verbal representations and processing, such as left lateral temporal cortex and inferior frontal cortex, and upon brain regions that underlie spatial representations and processing, such as right intra-parietal sulcus (IPS), precuneus, and posterior superior parietal cortex (Lee, 2000; Dehaene et al., 2003; Schmithorst and Brown, 2004; Andres et al., 2011; Prado et al., 2011; Menon, 2013). In the context of arithmetic processing, activation in verbal networks have been linked to retrieval of arithmetic facts and executive control (Prado et al., 2011), whereas activation in spatial networks have been linked to modality independent representations and procedural manipulation of numerical magnitude (De Smedt et al., 2011).

Although studies have found these regions to be engaged in most participants, activity within this network may vary as a function of task and children’s concurrent math skill (Zago et al., 2001; Grabner et al., 2007; Rosenberg-Lee et al., 2009; Cho et al., 2011; De Smedt et al., 2011). For example, De Smedt et al. (2011) showed that 10- to 12-year-old children with lower math skill activated the right intraparietal sulcus to a greater extent than children with higher skill for small addition and subtraction problems. The results were interpreted to suggest that low skill children might use procedural strategies (and rely on spatial neural representations), whereas higher skill children might retrieve arithmetical information from memory (and rely on verbal neural representations).

How the neural differences relate to growth in arithmetic skills is unclear. Some studies have shown that structural and intrinsic functional connectivity predicts math gains (Evans et al., 2015; Jolles et al., 2015). For example, a recent study reported that short-term arithmetic skill gains (8 weeks) after an intervention could be predicted by (1) gray matter volume in the hippocampus and (2) functional connectivity between hippocampus and dorsolateral and ventrolateral prefrontal cortices (as well as basal ganglia), highlighting the role of the hippocampus and memory in the development of arithmetic skills (Supekar et al., 2013). In a longitudinal study, improvement in arithmetic retrieval fluency over a 1-year period was related to hippocampus-neocortical connectivity (Qin et al., 2014). Here, for the first time, we examine the task-based functional neural predictors of long-term change in math skill, i.e., up to 3 years. Importantly, our main focus is on if the neural predictors of math skill growth vary along the SES gradient.

SES-related differences in mathematics are larger on verbal aspects of mathematics, such as verbally presented number combinations, than on spatial aspects, such as non-verbal calculations with disks (Jordan and Levine, 2009). SES-related differences in children’s verbal skills are well described and appear to be more robust than differences in spatial skills in other domains as well (Hart and Risley, 1995; Noble et al., 2007). In a recent neuroimaging study, we showed that the neural underpinnings of arithmetic processing vary as a function of SES and children’s concurrent math skill level (Demir et al., 2015). Reliance on brain regions that support verbal representations (i.e., middle temporal gyrus, MTG) was related to concurrent math skill to a greater extent for higher than lower SES children. On the contrary, reliance on brain regions that support spatial representations (i.e., IPS) were related to concurrent math skill to a greater extent for lower than higher SES children. Importantly, these differences were observed in a sample where a normative range of parental SES was represented. These results suggest that depending on their parental SES, children might develop adaptations and recruit alternative neural networks to perform at par with their peers.

This previous study left open the question of whether the neural networks that predict growth prospectively vary as function of SES and whether these are the same networks that are concurrently predictive of skill? In the present study, we asked how children’s early reliance on verbal and spatial neural systems during elementary arithmetic predicts math skill change, and importantly whether the neural systems that predict change vary as a function of SES. To address these questions, we measured brain activity of 8- to 13-year-old children during a single-digit subtraction task, as well as during verbal and spatial localizer tasks. We administered a standardized behavioral measure of math skill before scanning and up to 3 years later (Woodcock et al., 2001). We measured parental SES with parental education and occupation information. We specifically tested if in line with our previous findings, reliance on verbal neural systems would predict math skill growth for higher SES children, whereas reliance on spatial neural systems would predict math skill growth for lower SES children.

We used functional localizer tasks to identify the reliance on brain systems underlying verbal and spatial mechanisms during subtraction. We used a word rhyming task as our verbal localizer and a non-symbolic, dot comparison task as our spatial localizer. Previous literature showed that this word rhyming task taps into verbal representations and successfully localizes verbal neural systems in left temporo-parietal and inferior frontal cortices (Booth, 2010; Prado et al., 2011, 2014). Previous literature showed that this dot comparison task taps into spatio-numerical representations and successfully localizes regions in right intraparietal sulcus, superior parietal lobule and precuneus (Prado et al., 2011, 2014). Importantly, performance on tasks similar to our verbal and spatial localizer tasks relate to mathematical skill, suggesting an overlap between the neural basis of our localizers and mathematical performance (Siegel and Linder, 1984; Hecht et al., 2001; Halberda et al., 2008; Simmons et al., 2008; Krajewski and Schneider, 2009; Piazza et al., 2010).

## Materials and Methods

### Participants

Forty-one children were recruited from schools in the greater Chicago area to participate in the study^1^. All children (1) were native English speakers, (2) were free of past or present neurological or psychiatric disorders, (3) had no history of reading, oral language, or attention deficits, and (4) scored higher than 80 standard score on full scale IQ as measured by Wechsler Abbreviated Scale of Intelligence (WASI; Weschler, 1999). Data from eight participants were excluded because of excessive movement in the scanner (see criteria below, *n* = 6), low behavioral accuracy in the scanner (i.e., lower than 40% in the arithmetic and localizer tasks) and/or response bias in the scanner (i.e., false alarm to misses ratio greater than 2 and false alarm rate greater than 50%, *n* = 2). The remaining 33 participants (20 females) were included in the analyses. At the beginning of the study (T1) children were from 8 to 13 years of age (mean age = 10.9, *SD* = 1.5, range = 8–13.8). At the second visit (T2), children were from 11 to 16 years of age (mean age = 13.4, *SD* = 1.5, range = 10.6–16.1). Written consent was obtained from the children and their parents/guardians. All experimental procedures were approved by the Institutional Review Board at Northwestern University.

### Standardized Measures

Children were administered standardized measures to assess their intellectual and mathematical abilities on entering the study (T1) and after a follow-up period of 2.5 years (*SD* = 0.16, range = 2.2–2.8) (T2). We measured IQ by the Verbal (Vocabulary, Similarities) and Performance (Block Design, Matrix Reasoning) subtests of the WASI (Weschler, 1999). Mathematical skill was assessed by the Math Fluency subtest of the Woodcock-Johnson III Tests of Achievement (WJ-III, Woodcock et al., 2001). The Math Fluency subtest requires children to solve as many simple addition, subtraction, and multiplication problems as possible within a 3-min period. The difference in raw score between T1 and T2 was 21 points (*SD* = 14.2). **Table 1** summarizes children’s performance on standardized tests at T1 and T2.

**Table 1:** Means and SDs for behavioral measures.

	T1		T2	
		
	Mean	*SD*	Mean	*SD*
IQ (Standardized)	118.1	13.8	120.7	14.1
Math fluency (Standardized)	97.1	15.0	94.1	13.9
Math fluency (Raw)	65.8	24.7	85.7	25.2
Subtraction accuracy	83.3%	17.3%	–	–
Subtraction RT	1172	295	–	–
Verbal localizer accuracy	85.5%	11.0%	–	–
Verbal localizer RT	1280	214	–	–
Spatial localizer accuracy	87.6%	11.9%	–	–
Spatial localizer RT	1051	204	–	–


### Socioeconomic Status

Parental SES information was collected on entering the study (T1). A widely-used measure of SES, the Hollingshead Index, based on primary caregiver education and occupation was used as our measure of child SES (Hollingshead, 1975; Adams and Weakliem, 2011). The education level of the primary caregivers was measured categorically with values ranging between 1 (less than 7th grade) to 7 (graduate degree). The average Hollingshead education score for our sample was 6 (*SD* = 0.8), with a range from 4.5 to 7 years, corresponding to a college or associates degree. The occupation level of the primary caregivers was measured categorically with values ranging between 1 (farm laborer, menial service worker, student, housewife) and 9 (higher executive, large business owner, major professional). The average Hollingshead occupation score was 6 (*SD* = 2.3), with a range from 1 to 9, corresponding to technician, semi-professional or small business owner. Following Hollingshead, SES was calculated using the formula (Occupation × 7) + (Education × 4), (*M* = 50.9, *SD* = 14.3). For 24 children both mother and father were primary caregivers, whereas for nine children mother was the primary caregiver. For children with dual caregivers, average education and highest occupation level was used. For the remaining, the education and occupation information of the primary caregiver was used. Average primary caregiver education and occupation were highly correlated with each other, *r* = 0.74, *p* < 0.04.

### Arithmetic Task

**FIGURE 1:**
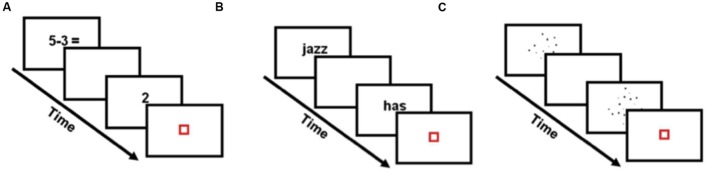
**Experimental tasks.** **(A)** In the arithmetic problems, participants were asked to evaluate subtraction problems. Localizer tasks were used to identify the Regions of Interest. **(B)** In the verbal localizer task, participants decided if two words rhymed or not. **(C)** In the spatial localizer task, participants decided which dot array included a larger number of dots.

Children were administered a single-digit subtraction task in the scanner on entering the study (T1). In each trial of the subtraction task, children were asked to evaluate whether the answer to a single-digit subtraction problem was true or false (**Figure 1A**). Twenty-four number pairs were used, covering the full range of single-digit subtraction problems (with the exceptions below). Each pair was repeated twice with a true answer (e.g., 5 – 3 = 2) and once with a false answer. Thus, children were presented with 72 problems in the main experiment and 24 problems in the practice session. False answers were created by subtracting 1 from the correct answer (e.g., 5 – 3 = 1) or by adding 1 or 2 to the correct answer (e.g., 5 – 3 = 4). Problems with 0 or 1 as the second operand (e.g., 5 – 0), tie problems where the first and second operand are identical (e.g., 5 – 5), problems where the correct answer correspond to the second term (e.g., 6 – 3) and problems where the first operand is smaller than the second (e.g., 3 – 5) were not used.

### Localizer Tasks

Children were administered two localizer tasks in the scanner on entering the study (T1). We used a word rhyming task to localize verbal neural systems. In each trial of the verbal localizer, two words were sequentially presented. Children were asked to evaluate whether the two words rhymed or not (**Figure 1B**). All words were monosyllabic English words with varying orthographic and phonological similarity (e.g., dime – lime, pint – mint, grade – laid, press – list). Similarity was manipulated so that responses could not be based on spelling alone. Fouty-eight word pairs were used in the main experiment (24 similar, 24 not similar) and 48 word pairs were used in the practice session. We used a non-symbolic, dot comparison task to localize brain regions that subserve spatial representations. In each trial of the spatial localizer, two dot arrays were sequentially presented (**Figure 1C**). Children were asked to decide which of the two dot arrays were composed of a larger number of dots. Arrays of 12, 24, and 36 dots were used with varying single dot sizes and cumulative surface area. Seventy-two pairs of dot arrays were used in the main experiment and 36 pairs were used in the practice session. **Table 1** summarizes children’s performance on subtraction and localizer tasks.

### Experimental Procedure

At T1, after informed consent was obtained and standardized tests were administered, children participated in a practice session. During the practice session, children learned to minimize their head movement (with feedback from an infrared tracking device), and practiced all three tasks in a mock fMRI scanner. The actual fMRI scanning session took place within one week of the practice session. In the fMRI scanner, subtraction and spatial localizer tasks were divided into two runs of about 4 min each. The verbal localizer task was administered in a single run lasting about 7 min. The order of tasks was counterbalanced across participants. Behavioral responses were recorded using an MR-compatible keypad placed below the right hand. Visual stimuli were generated using E-prime software (Psychology Software Tools Inc., 2012), and projected onto a translucent screen. Children viewed the screen through a mirror attached to the head coil.

Stimulus timing was identical in all tasks. A trial started with the presentation of a first stimulus (subtraction, dot array or word depending on the task) for 800 ms, followed by a blank screen for 200 ms. A second stimulus (subtraction, dot array or word depending on the task) was presented for 800 ms, followed by a red fixation square presented for 200 ms. Participants were asked to make a response during an interval ranging from 2,800 ms to 3,600 ms. Twenty-four null trials were included in the subtraction and spatial localizer tasks. Twelve null trials were used for the verbal localizer task. In the null trials, a blue square was presented for the same duration as the experimental conditions and children were asked to press a button when the square turned red. Each run ended with 22 s of passive visual fixation. Fixation periods (between trials and at the end of the run) constituted the baseline. The timing and order of trial presentation within each run was optimized for estimation efficiency using Optseq2^2^ (Dale, 1999).

### fMRI Data Acquisition

Images were collected using a Siemens 3T TIM Trio MRI scanner (Siemens Healthcare, Erlangen, Germany) at Northwestern University’s Center for Translational Imaging (CTI). The fMRI blood oxygenation level-dependent (BOLD) signal was measured with a susceptibility weighted single-shot echo planar imaging (EPI) sequence. The following parameters were used: TE = 20 ms, flip angle = 80°, matrix size = 128 × 120, field of view = 220 mm × 206.25 mm, slice thickness = 3 mm (0.48 mm gap), number of slices = 32, TR = 2,000 ms. Before functional image acquisition, a high resolution T1-weighted 3D structural image was acquired for each subject (TR = 1,570 ms, TE = 3.36 ms, matrix size = 256 × 256, field of view = 240 mm, slice thickness = 1 mm, number of slices = 160).

### Behavioral Data Analyses

The math change score was calculated by subtracting children’s raw score on the Math Fluency subtest at T1 from their score at T2 and dividing this change score by the age difference between T2 and T1^3^. This measure reflected the rate of change in math score between T1 and T2. In order to examine if any of the behavioral measures collected at T1 predicted math change score, math change score was correlated with IQ, Math Fluency score (raw and standardized score), subtraction accuracy and RT, and age at T1. Correlations with parental SES were also calculated.

### fMRI Data Analyses

Data analyses were performed using SPM8 (Statistical Parametric Mapping^4^). The first six images of each run were discarded, functional images were corrected for slice acquisition delays, realigned to the first image of the first run to correct for head movements, and spatially smoothed with a Gaussian filter equal to about twice the voxel size (4 mm × 4 mm × 8 mm full width at half maximum). ArtRepair software was used to suppress residual fluctuations due to large head motion and to identify volumes with significant artifact and outliers relative to the global mean signal (4% from the global mean). Volumes showing rapid scan-to-scan movements of greater than 1.5 mm were excluded via interpolation of the two nearest non-repaired volumes. Interpolated volumes were partially deweighted when first-level models were calculated on the repaired images (Mazaika et al., 2009). All participants had less than 5% of the total number of volumes replaced in a single run. Average translation and rotation movements were small (*x*-plane: *M* = 0.06 mm; *y*-plane: *M* = 0.08 mm, *z*-plane: *M* = 0.27 mm, pitch: *M* = 0.27°, roll: *M* = 0.12°, yaw: *M* = 0.09°). Functional volumes were co-registered with the segmented anatomical image and normalized to the standard T1 Montreal Neurological Institute (MNI) template volume (normalized voxel size, 2 mm × 2 mm × 4 mm).

#### First Level Analyses

Event-related statistical analyses were performed according to the General Linear Model. Activation was modeled as epochs with onsets time-locked to the presentation of the first stimulus (operands) and ending at the offset of the second stimulus (answer). For the arithmetic tasks, all responses were included in the model. However, only responses in problems with a true answer were considered of interest in the analyses to avoid inhibitory processes associated with rejecting invalid trials. All epochs were convolved with a canonical hemodynamic response function. The time series data were high-pass filtered (1/128 Hz), and serial correlations were corrected using an autoregressive AR(1) model. Effect sizes were estimated using linear statistical contrasts and subsequently entered into second level analyses.

#### Second Level Analyses

In order to evaluate the relations between SES, rate of math score change and neural bases of arithmetic, second level voxel-wise regression models were created. In each analysis, SES, math change score, as well as the interaction between SES and math change score constituted the regressors of interest. Additionally, we included as regressor of no interest full scale IQ at T1. Our specific question was about interactive relations of math change score and parental SES to the neural basis of arithmetic. We identified brain regions that showed an increase or a decrease in activity during the evaluation of subtraction problems with respect to the interaction term across subjects. All analyses were repeated with measures of performance (accuracy) on the arithmetic task and T1 math score as regressors of no interest and the results reported below remained unchanged, as described below. We specifically focused on the interaction between math change score and SES because of the nature of our specific question and also in order to reduce our Type 1 error. Analyses examining main effects of math change score and SES on the neural basis of arithmetic are provided in Supplementary Materials.

#### ROI Definition

The relations of SES and rate of math change score to the neural basis of subtraction were examined within verbal and spatial ROIs. Verbal ROIs were identified using the verbal localizer contrast (contrast of [words versus null trials] across all subjects). Spatial ROIs were identified using the spatial localizer contrast (contrast of [dots versus null trials]). The resulting statistical maps were thresholded for significance using a voxelwise threshold of *p* < 0.01 (uncorrected) and a clusterwise threshold of *p* < 0.05 (FWE corrected for multiple comparisons). To ensure the specificity of the localizer activation (i.e., no overlap between localizers), each contrast was exclusively masked by the voxels in which the other localizer contrast was positive (exclusive mask thresholded at *p* < 0.05 uncorrected).

**FIGURE 2:**
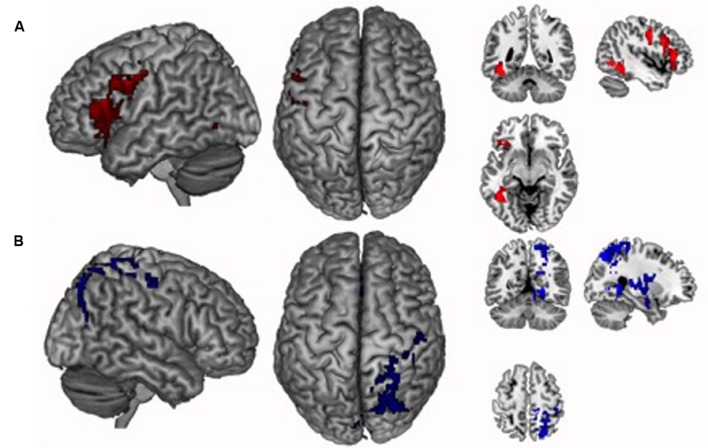
**Brain networks identified by the localizer tasks.** **(A)** The verbal localizer task was associated with enhanced activity in a network that included left inferior/middle temporal, inferior/middle frontal, fusiform and precentral gyri. **(B)** The spatial localizer task was associated with enhanced activity in a network that included right inferior/superior parietal lobule, precuneus, cuneus, posterior cingulate, lingual gyrus, postcentral gyrus, insula, putamen and left anterior cingulate. Activations are overlaid on a 3D rendering and on coronal, sagittal, and axial slices of the MNI-normalized anatomical brain.

The verbal localizer contrast was associated with enhanced activity in the left inferior/middle temporal, inferior/middle frontal, fusiform, and precentral gyri (**Figure 2A** and **Table 2**). These clusters constitute the verbal localizer mask. The spatial localizer contrast was associated with enhanced activity in multiple clusters spanning right inferior/superior parietal lobule, precuneus, cuneus, posterior cingulate, lingual gyrus, postcentral gyrus, insula, putamen and left anterior cingulate (**Figure 2B** and **Table 2**). These clusters constituted the spatial localizer mask. The localizers enabled us to independently identify brain regions that subserve verbal versus spatial processes.

**Table 2:** Peak activated voxels in the localizer tasks.

Anatomical location	∼BA	MNI coordinates			*Z*-score	Size
				
		X	Y	Z		
L. inferior/middle temporal gyrus/fusiform gyrus	19/37/39	-44	-60	-6	5.51	292
L. inferior/middle frontal gyrus/precentral gyrus	6/45/46	-50	-6	38	4.62	916
**Spatial localizer**						
R. cuneus/posterior cingulate/lingual gyrus	17/18/30	14	-78	6	4.75	520
R. superior parietal lobule/precuneus/postcentral gyrus	5/7/31	28	-48	62	4.50	806
R. inferior parietal lobule/insula/putamen	13/40	42	-24	26	5.32	997
L. anterior cingulate	32	-12	26	30	4.25	350


*L, left; R, right; ∼BA, approximate Brodmann area for the peak coordinate; MNI, Montreal Neurological Institute; Size, number of 2 mm × 2 mm × 4 mm voxels.*

#### ROI Analyses

Statistical significance within each of these localizer masks was defined using Monte Carlo simulations (using AFNI’s AlphaSim program^5^). In order to reach corrected level threshold (alpha = 0.05) within the verbal ROIs, the clusters needed to contain 75 voxels with a height threshold of 0.05. Within the spatial ROIs, clusters needed to contain 85 voxels with a height threshold of 0.05. Statistical maps were used to estimate smoothness. Throughout the paper, we consider a cluster significant if *p* < 0.05 and a trend if *p* < 0.1.

#### Whole Brain Analyses

To investigate non-predicted effects in regions outside verbal or spatial ROIs, we also report results of whole-brain analyses conducted outside the ROIs reported above. The statistical maps were thresholded for significance using a voxelwise threshold of *p* < 0.01 (uncorrected) and a clusterwise threshold of *p* < 0.05 (FWE corrected for multiple comparisons).

## Results

### Behavioral Performance and Relations to Math Change Score

**Table 3** summarizes correlations between behavioral measures at T1 (e.g., IQ, math score), math change score and SES. None of the behavioral measures significantly predicted change in math scores. We included IQ as a covariate in our analyses, but results remained unchanged using other covariates as described below. SES did not significantly relate to any of the measures at T1 or to change in math score. In a series of regression analyses, we examined whether math score change was related to the interaction of SES with any of the behavioral measures. We included SES, behavioral measures and their interaction as independent variables and math change score as the dependent variable. None of the interaction terms predicted math score change.

**Table 3:** Correlations between SES, behavioral measures at T1, and change in math fluency.

T1	Change in math fluency	SES
SES	0.01	–
Change in math fluency	–	0.01
Age	-0.18	–0.23
WASI IQ (Standardized)	0.22	0.13
WJ math fluency (Standardized)	-0.06	0.10
WJ math fluency (Raw)	-0.24	0.24
Subtraction accuracy	-0.06	0.09
Subtraction RT	-0.09	0.23
Verbal localizer accuracy	0.12	0.24
Verbal localizer RT	-0.09	0.11
Spatial localizer accuracy	0.09	–0.10
Spatial localizer RT	0.08	–0.06


*None of these correlations were significant.*

### Overall Activation in Verbal and Spatial ROIs during the Subtraction Task

We first examined overall activation in the verbal and spatial ROIs during the subtraction task, using the contrast of [subtraction trials– baseline] submitted to a one-sample *t*-test across all participants. In verbal ROIs, subtraction problems showed significant activation in left IFG (peak coordinate: *x* = -52, *y* = 8, *z* = 38, BA = 9, *z* = 4.21, *k* = 324 voxels) and in left MTG (peak coordinate: *x* = -44, *y* = -60, *z* = -6, BA = 21, *z* = 3.71, *k* = 178 voxels). In spatial ROIs, subtraction problems showed activation in right culmen/lingual gyrus (peak coordinate: *x* = 10, *y* = -62, *z* = -14, BA = 19, *z* = 5.52, *k* = 109 voxels), and although not significant, subtraction problems also showed activation in precuneus (peak coordinate: *x* = 26, *y* = -46, *z* = -46, BA = 7, *z* = 3.12, *k* = 51 voxels).

### Relation between Change in Math Score and Neural Activity during the Subtraction Task is Moderated by Parental SES

We then examined whether SES moderates the relation of rate of math score change to the neural basis of subtraction problems. We identified the brain regions within our verbal or spatial ROIs where activity during the evaluation subtraction problems was associated with the interaction between SES and math change score (when the effects of IQ were controlled).

#### Verbal ROIs

**FIGURE 3:**
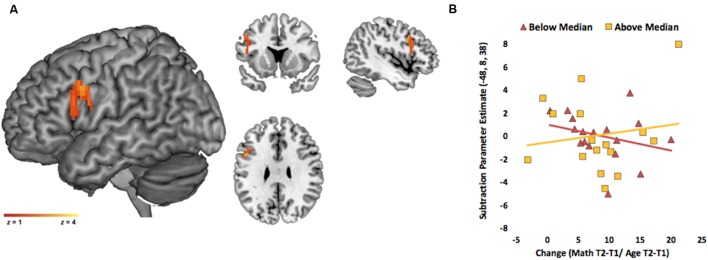
**Interaction between SES and math score change in the verbal ROI for subtraction problems.** **(A)** Activity in left inferior frontal gyrus (IFG) showed a SES and change interaction. Activation is overlaid on a 3D rendering and on coronal, sagittal, and axial slices of MNI-normalized anatomical brain. **(B)** Average brain activity (adjusted eigenvariates) was extracted from the significant cluster in left IFG and plotted against change scores for visualization purposes only. Relation was visualized for children above the median SES and below the median SES.

We found a significant interaction (SES × change) in a cluster in left IFG (peak coordinate: *x* = -48, *y* = 8, *z* = 34, BA = 9, *z* = 3.18, *k* = 79 voxels; **Figure 3A**)^6^. For visualization purposes only, we divided the children into two groups based on median SES (lower than or at the median constituting lower SES, and higher than the median constituting higher SES). We then extracted the adjusted eigen variate from the significant cluster and plotted it against math change score for the two SES groups. This plot showed that for higher SES, change is positively associated with activity during subtraction in left IFG, but the relation is negative for lower SES (**Figure 3B**).

Finally, the interaction identified with continuous variables was confirmed with follow-up analyses comparing relations between change score and activation for higher versus lower SES children. For these analyses, we divided the children into two groups based on median SES (lower than or at the median constituting lower SES, and higher than the median constituting higher SES). We conducted a full factorial design including SES as a binary variable (higher, lower), change as continuous variable, as well as an interaction term between the binary SES variable and change. IQ was included as a continuous covariate. The interaction term enabled us to directly compare the association between change and brain activity in higher versus lower SES. We first identified areas in verbal ROIs where brain activity was associated with change to a greater extent for higher than lower SES children. This direct comparison revealed that a cluster in left IFG was significantly and more strongly related to change in higher SES children than lower SES children (peak coordinate: *x* = -50, *y* = 8, *z* = 34, BA = 9, *z* = 3.56, *k* = 187 voxels). This cluster overlaps with the cluster identified by the analyses using the continuous variables. The reverse contrast did not reveal any significant activation – there were no significant clusters in verbal ROIs where activation was related to change more strongly for lower than higher SES children.

#### Spatial ROIs

**FIGURE 4:**
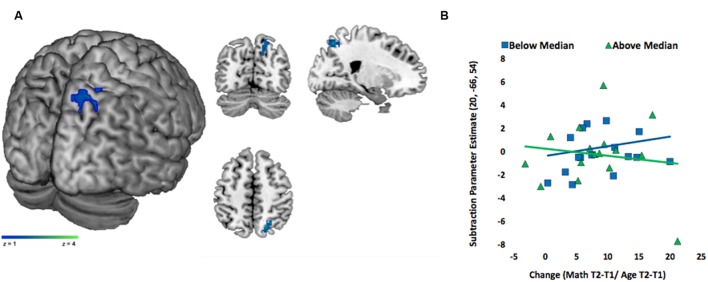
**Interaction between SES and math score change in the spatial ROI for subtraction problems.** **(A)** Activity in right superior parietal sulcus/precuneus (PSPL/Pr) showed a SES and change interaction. Activation is overlaid on a 3D rendering and on coronal, sagittal, and axial slices of MNI-normalized anatomical brain. **(B)** Average brain activity (adjusted eigenvariates) was extracted from the significant cluster (3 mm around the peak) in right PSPL/Pr, and plotted against change scores for visualization purposes only. Relation was visualized for children above the median SES and below the median SES.

We found a marginally significant interaction in a cluster in right PSPL/Pr, (peak coordinate: *x* = 20, *y* = -66, *z* = 54, BA = 7, *z* = 2.52, *p* = 0.06, *k* = 83 voxels; **Figure 4A**)^7^. For visualization purposes only, we divided the children into two groups based on median SES. We then extracted the adjusted eigenvariate from the significant cluster and plotted it against math change score for the two SES groups. This plot showed that for lower SES children, change is positively associated with activity during subtraction in right PSPL/Pr, but the relation between change and activation in this area is negative for higher SES children (**Figure 4B**).

In order to confirm the interaction identified with continuous variables, we then divided the children into two groups based on median SES (lower than or at the median constituting lower SES, and higher than the median constituting higher SES). We conducted a full factorial design including SES as a binary variable (higher, lower), change as continuous variable, as well as an interaction term between the binary SES variable and change. IQ was included as a continuous covariate. The interaction term enabled us to directly compare the association between change and brain activity in higher versus lower SES. We identified areas in spatial ROIs where brain activity was associated with change to a greater extent for lower than higher SES children in spatial ROIs. This direct comparison revealed that a cluster in right PSPL/Pr was significantly and more strongly related to change in lower SES children than higher SES children (peak coordinate: *x* = 20, *y* = -66, *z* = 54, BA = 7, *z* = 2.96, *k* = 95 voxels). This cluster overlaps with the cluster identified by the analyses using the continuous variables. The reverse contrast also revealed a significant activation in the spatial ROIs– a cluster in inferior parietal, specifically extending from postcentral gyrus to insula where activation was related to change more strongly for higher SES than lower SES children (peak coordinate in the insula: *x* = 30, *y* = -30, *z* = 18, BA = 13, *z* = 2.70, *k* = 250 voxels).

#### Whole Brain Analyses

Outside the ROIs, the interaction term (SES × change) was significantly and positively related to activation in two clusters. Activity in these clusters was related to greater growth in math skill at the higher end of the SES continuum, but lesser improvements at the lower end. The two clusters included one spanning right supramarginal, superior temporal and extending into inferior parietal gyri (peak coordinate, *x* = 42, *y* = -44, *z* = 22, BA = 13/22/40, *z* = 3.81, *k* = 438 voxels) and another in right inferior frontal, middle frontal, and extending into precentral gyri (peak coordinate, *x* = 46, *y* = 10, *z* = 30, BA = 6/9, *z* = 3.58, *k* = 278 voxels). The latter peak was within 5 mm of the peak noted above identified within the verbal ROIs in the left hemisphere. There were no areas that were negatively and significantly related to the interaction term outside the ROIs.

## Discussion

Children differ widely in their math skill growth, and parental SES is one of the strongest predictors of these individual differences. To our knowledge, nothing is known about how early neural predictors of later math skill growth vary for children at different SES levels. In the current study, we independently identified brain regions that subserve verbal and spatial neural systems using localizer tasks. We asked how early reliance on these regions relate to growth in math skill over a 3-year period and whether the neural predictors vary as a function of parental SES. Results showed that early neural predictors of math skill gains encompassed brain regions underlying verbal processing, such as left inferior frontal and middle temporal gyri, as well as visuo-spatial processing, such as right culmen/lingual gyrus and precuneus. In addition, neural predictors of math gains varied depending on parental SES.

Activity in an area of the left inferior frontal gyrus (IFG) identified by the verbal localizer was related to greater growth in math skill at the higher end of the SES continuum, but lesser improvements at the lower end. We showed that early reliance on verbal neural systems, specifically left IFG, predicted rate of change in math skill to a greater extent for children at the higher end of the SES continuum than the lower end. Left IFG is consistently activated in arithmetic tasks, and considered to be involved the manipulation of verbal representations of arithmetic rules and facts hosted in left middle temporal cortex (Kucian et al., 2008; Rosenberg-Lee et al., 2009). Left IFG is also implicated in executive control, which is strongly associated with both SES and arithmetic skill (Bull and Scerif, 2001; Badre and Wagner, 2007; Hackman and Farah, 2009).

During development higher SES children might have learned to better manipulate verbal representations in general and verbal representations of numerical quantities more specifically (for example between Arabic numbers and their meanings) as compared to lower SES children. Differential relations of SES and math skill growth in left IFG might reflect more robust manipulation of such verbal representations by higher SES with higher skill growth children compared to children from lower SES backgrounds. This might aid higher SES children when learning arithmetic problems, e.g., in forming associations between problems and answers or acquiring arithmetic rules and procedures, more than lower SES children. Whole brain analyses revealed right-lateralized activation in right supramarginal/superior temporal and inferior/middle frontal areas to be more strongly associated with math skill change at the higher end of the SES continuum as compared to the lower end. A bilateral fronto-temporal network including these regions has been argued to underlie verbal processing of arithmetic problems, specifically of verbal retrieval or verbalization (Zarnhofer et al., 2012). Thus, whole-brain analyses add support to the interpretation that reliance on verbal representations might predict growth in math skill in higher SES to a greater extent than lower SES children.

In a previous paper, we showed that, at higher levels of SES, higher math skill was associated with concurrent reliance on left MTG, but not IFG (Demir et al., 2015). Left temporo-parietal cortices are thought to support verbal representations, such as representations of the associations between arithmetic problems and their solutions (Booth et al., 2002; Fiebach et al., 2002; Blumenfeld et al., 2006; Prado et al., 2011). Combined with the current findings, these results suggest that concurrent math skill might be related to the representational systems themselves hosted in middle temporal regions, whereas acquiring new math knowledge might be associated with ‘higher-level’ regions manipulating these representations, such inferior frontal regions.

Early parental input might explain why reliance on verbal neural systems predicts growth in math skill to a greater extent for higher than lower SES children. Children differ widely from each other along the SES continuum in their exposure to verbal input in general and verbal input about mathematics specifically, but SES differences in exposure to spatial stimulation are less consistent (Saxe et al., 1987; Hart and Risley, 1995; Blevins-Knabe and Musun-Miller, 1996; Hoff, 2003; Ehrlich, 2007; Levine et al., 2010; Gunderson and Levine, 2011; Levine et al., 2012). The quantity of parental number talk during naturalistic parent–child interactions during preschool years is higher in higher SES families (Gunderson and Levine, 2011). Parental verbal input strongly relates to preschool numerosity outcomes, more strongly than numerosity-related activities (Gunderson and Levine, 2011; Anders et al., 2012). Previous neuroimaging studies suggest that the neural basis of verbal processing, specifically left IFG, is more specialized in higher SES children, confirming our findings regarding left IFG predicting greater change for higher SES children than lower SES children (Pakulak et al., 2005; Raizada et al., 2008; Hackman and Farah, 2009).

Activity in an area of the right superior parietal cortex identified by the spatial localizer was related to greater growth in math skill at the lower end of the SES continuum, but lesser improvements at the higher end. Early reliance on spatial neural systems, specifically right superior parietal cortex/precuneus (PSPL/Pr), predicted rate of change in math skill to a greater extent for lower than higher SES children. The right PSPL/Pr is considered to be involved in spatial and attentional processes and, in the context of arithmetic, in the spatial manipulation of numerical magnitudes, hosted in right intraparietal sulcus (Dehaene et al., 2003; Ischebeck et al., 2006; Metcalfe et al., 2013; Prado et al., 2014; Berteletti et al., 2015). We argue that differential relations of SES and math skill growth in right PSPL/Pr might reflect more robust manipulation of spatial representations of numbers by children with lower SES with higher skill growth. In the absence of the rich verbal input that higher SES children receive, lower SES children might rely on spatial strategies in learning arithmetic to a greater extent than higher SES children. It should be noted that the interaction effect we observed might also be due to higher SES children showing a negative relation to change in right PSPL/Pr – children who use spatial strategies despite being exposed to rich input might exhibit shallower growth over time.

Indeed, SES-related differences in mathematical cognition tend to be larger on verbal aspects of math as compared to spatial aspects (Jordan and Levine, 2009). Interventions aiming to improve mathematical cognition in low SES children are also reported to improve performance on verbal aspects of math, e.g., comparison of number words, but not non-symbolic, spatial aspects, e.g., comparison of magnitudes, suggesting greater room for growth in verbal systems (Wilson et al., 2009). Extending our findings to the domain of reading, Gullick et al. (in press) recently similarly reported that the relation of reading skill to white matter depends on SES. For lower SES children, higher reading skill was correlated with white matter in right hemisphere visuo-spatial tracts, suggesting that lower SES children may rely more on visuo-spatial orthographic processing strategies for reading success. Thus, lower SES children might find rely on visuo-spatial neural systems to a greater extent than higher SES children across different academic tasks.

Prior literature suggested that SES-related differences in mathematics are larger on verbal aspects of mathematics than on spatial aspects (Jordan and Levine, 2009). In general, SES-related differences in children’s verbal skills are well described and appear to be more robust than differences in spatial skills (Hart and Risley, 1995; Noble et al., 2007). Our findings add to the existing literature suggesting that the nature of SES differences might be better described as interacting with children’s skill and highlight differential relationships between SES and verbal versus spatial neural systems, rather than an overall effect of SES on verbal systems. In sum, depending on parental SES, children might develop adaptations and recruit alternative neural networks to varying degrees to perform at par with their peers.

In a previous study (Demir et al., 2015), we showed the activation in right IPS to relate to concurrent math skill for children at the lower end of the SES continuum. The IPS has been argued to house spatial representations important for arithmetic processing (Dehaene et al., 2003). These results combined with current findings support our argument regarding the distinction between neural systems that support representations themselves for concurrent performance versus manipulation of these representations for learning. Indeed, longitudinal behavioral studies with children showed that working memory is a strong predictor of mathematical skill growth over and above the contributions of domain-specific quantitative, calculation or reading skill, short-term memory and phonological processing skill (Bull et al., 2008; Swanson et al., 2008; Welsh et al., 2010; Metcalfe et al., 2013). Similarly, a recent neuroimaging study found that activation in parietal lobule during a visuo-spatial working memory task predicts math skill growth over a 2-year period (Dumontheil and Klingberg, 2011).

Our results showed that growth in math skill was significantly predicted by neural, but not behavioral measures included in the study, e.g., IQ, early math skill and age. Although null results are hard to interpret, these results are in line recent neuroimaging studies in math and reading development that showed predictive power of neural differences over and above behavioral differences (Hoeft et al., 2007, 2011; McNorgan et al., 2011; Supekar et al., 2013). Neuroimaging measures might serve as sensitive measures of individual differences in underlying neural mechanisms not fully captured by current behavioral standardized tests. This highlights the possibility of using early neural markers to predict future math performance.

The current study raises various questions to be addressed by future research. First, the current study specifically focused on subtraction problems. Prior studies have shown that subtraction problems activate both verbal and visuo-spatial neural systems, and thus subtraction problems might be more appropriate to examine the differential reliance of SES on verbal versus spatial neural systems (Siegler, 1988; De Smedt et al., 2011). However, future studies should examine SES relations to the neural basis of other operations, specifically those that primarily rely on verbal representations, such as multiplication (Lee and Kang, 2002; Prado et al., 2013). Second, our study did not include children at lowest end of the SES continuum. This enabled us to examine SES-related differences within the normative range of SES, in the absence of other confounding factors, such as nutritional differences, differences in sleep patterns or stress. In our study children’s behavioral performance on single-digit arithmetic problems did not vary according to SES, which also allowed us to examine SES-related differences without confounding neural effects with differences due to accuracy or motivation. Future studies should examine SES-related differences in neural predictors of growth in more complex mathematical tasks where SES discrepancies are particularly wide, such as math word problems and on a wider SES continuum (Abedi and Lord, 1998; Jordan and Levine, 2009). Third, SES is a broad measure encompassing multiple characteristics including parental education, occupation, income, perceived social status, and is associated with parental cognitive stimulation, access to education, high-quality neighborhoods, and reduced stress among others (Brooks-Gunn and Duncan, 1997; Bradley and Corwyn, 2002; Hackman and Farah, 2009; Duncan and Magnuson, 2012). We used widely used indicators of SES that strongly relate to academic outcomes and we controlled for effects of IQ to gain more specificity about SES effects. Future studies should provide further specificity regarding the relations of different components of SES and neural basis of arithmetic development. Finally, it is important highlight that neural predictors of math growth encompassed brain regions that underlie both verbal and spatial processing. It was the relative degree to which activity in an area was related to math gains that varied along the SES continuum. Future longitudinal studies should focus on when do the differences along the SES gradient emerge and develop over time.

In summary, we, for the first time, highlight how neural systems that may be early neural predictors of long-term mathematical learning vary as a function of SES. Reducing the achievement gap necessitates a nuanced understanding of children’s differences early on. Although many intervening steps still need to be taken, targeted interventions that build upon early neural indicators might effectively address the challenges of children from differing backgrounds.

## Author Contributions

JB directed the larger study in which the present sub-study is embedded. OD-L, JP, and JB substantially contributed to the sub-study conceptualization and design of the work. JB and JP developed the experimental paradigm. OD-L and JP oversaw data collection. OD-L conducted the data processing, data analysis, and interpretation with input from JP and JB. OD-L drafted the manuscript, and all authors provided critical revisions. All authors approved the final version of the manuscript for submission. All authors agree to be accountable for all aspects of the work in ensuring that questions related to the accuracy or integrity of any part of the work are appropriately investigated and resolved.

## Conflict of Interest Statement

The authors declare that the research was conducted in the absence of any commercial or financial relationships that could be construed as a potential conflict of interest.

## Footnotes

1 Fifteen of the children included in the current study overlapped with the previous Demir et al. (2015) study examining concurrent relations between math skill and parental SES and neural basis of arithmetic performance.

2 http://surfer.nmr.mgh.harvard.edu/optseq/

3 We used raw scores rather than standardized scores because we were interested in the rate of growth in absolute arithmetic knowledge, rather than relative to peers. Standardized and raw change scores were significantly correlated with each other, *r* = 0.72, *p* < 0.001. Results remain unchanged using standardized scores.

4 www.fil.ion.ucl.ac.uk/spm

5 http://afni.nimh.nih.gov/

6 The pattern of results for the interaction term in the verbal ROI remained unchanged when controlling for accuracy on task (*z* = 3.23, *k* = 82) and math score at T1 (*z* = 3.32, *k* = 71).

7 The pattern on results for the interaction term in the spatial ROI remained unchanged when controlling for accuracy on task (*z* = 3.23, *k* = 66) and math score at T1 (*z* = 2.61, *k* = 92).
