# Supplementary material for: Neural Correlates of Math Gains Vary Depending on Parental Socioeconomic Status (SES)
Source: Front Psychol. 2016 Jun 17;7:892. doi: 10.3389/fpsyg.2016.00892 (PMC4911362; doi:10.3389/fpsyg.2016.00892)
Supplement: Supplementary file 1 [file Data_Sheet_1.DOCX]

**Supplementary analyses**

In order to examine the relations between math change score and the neural bases of arithmetic, we identified the brain regions that showed an increase or a decrease in activity during the evaluation of subtraction problems with respect to math score change across subjects. In order to examine the relations between SES and the neural bases of arithmetic, we identified the brain regions that showed an increase or a decrease in activity during the evaluation of subtraction problems with respect to SES across subjects.

**Relation between parent SES and neural activity during the subtraction task**

We identified the brain regions within our verbal or spatial ROIs where activity during the evaluation of subtraction problems was associated with SES (when the effects of IQ and math score change were controlled). The contrast of interest was [subtraction trials versus baseline]. There were no significant clusters within the verbal or spatial ROIs that were positively associated with SES. In spatial ROIs, SES was negatively associated with activation in right posterior superior parietal lobule/precuneus (PSPL/Pr) (peak coordinate: x = 10, y = -72, z = 34, BA = 7, *z* = 3.99, k = 314 voxels) and in right lingual gyrus (peak coordinate: x = 10, y = -54, *z* = 2, BA = 19, *z* = 3.37, k = 161 voxels). In verbal ROIs, SES was negatively associated with activation in left fusiform gyrus (peak coordinate: x = -34 y = -44, z = -18, BA = 37, *z* = 4.19, k = 166 voxels).

**Relation between change in math score and neural activity during the subtraction task**

We also identified brain regions within our verbal and spatial ROIs that showed a significant association with change (when effects of IQ and SES were controlled). There were no significant clusters that were positively related to change in verbal or spatial ROIs. In spatial ROIs, there was a negative trending relation between change and activation in right PSPL/Pr (peak coordinate: x = 14, y = -62, z = 30, BA = 31, *z* = 3.84, *p* = .08, k = 75 voxels).

**Whole brain analyses**

Outside the ROIs, SES was negatively related to activation in a large cluster spanning left fusiform gyrus, left middle occipital gyrus and right precuneus (peak coordinate, x =-34, y – 46, z =-18, BA = 7 / 19 / 37, *z* = 4.35, k =2883).
